# Metallomic profile in non-cirrhotic hepatocellular carcinoma supports a phenomenon of metal metabolism adaptation in tumor cells

**DOI:** 10.1038/s41598-021-93369-4

**Published:** 2021-07-09

**Authors:** Luis Cano, Stéphane Bertani, Marie-Laure Island, Juan Pablo Cerapio, Eloy Ruiz, Pascal Pineau, Valérie Monbet, Karim Boudjema, Luis Taxa, Sandro Casavilca-Zambrano, Martine Ropert, Bruno Turlin, Olivier Loréal

**Affiliations:** 1 INSERM UMR 1241, Nutrition, Metabolisms and Cancer Institute (NUMECAN), AEM2 Platform, INRAE, CHU Pontchaillou, University of Rennes, 2 Rue Henri le Guilloux, 35033 Rennes, France; 2 IRD, UPS, UMR152 Pharmadev, Université de Toulouse, Toulouse, France; 3 Centre de Recherches en Cancérologie de Toulouse, INSERM, UPS, UMR 1037, CNRS, ERL, Université de Toulouse, 5294 Toulouse, France; 4 Departamento de Cirugía Abdominal, Instituto Nacional de Enfermedades Neoplásicas, Lima, Peru; 5 Unité Organisation Nucléaire et Oncogenèse, INSERM, U993, Institut Pasteur, Paris, France; 6 IRMAR, INRIA, Université de Rennes, Rennes, France; 7 Service de Chirurgie Hépatobiliaire et Digestive, CHU Rennes, Université de Rennes, Rennes, France; 8 Departamento de Patología, Instituto Nacional de Enfermedades Neoplásicas, Lima, Peru; 9 Pathology Department, CHU Pontchaillou, Rennes University Hospital, 2 Rue Henri le Guilloux, 35033 Rennes, France

**Keywords:** Metals, Cancer metabolism, Liver cancer

## Abstract

We have previously described a form of hepatocellular carcinoma (HCC) in non-cirrhotic liver (HCC-NC) developed by Peruvian patients. We analyzed the metallomic profile in hepatic tissues from two independent cohorts exhibiting HCC-NC. Clinical, histopathological data, and HCC and non-tumoral liver (NTL) samples of 38 Peruvian and 38 French HCC-NC patients, were studied. Twelve metals were quantified using ICP/MS: Mn, Fe, Cu, Co, Zn, As, Se, Rb, Mo, Cd, Pb, and Sn. Associations between metals and survival were assessed. Our data showed significant differences between cohorts. Mean ages were 40.6 ± 20, 67.5 ± 9 years old for Peruvians and French, respectively. Fifty percent of the Peruvian patients were positive for the HBsAg, versus 3% in French patients. Mn, Cu, Zn, As, Se, Rb, Mo, Cd, Sn metal concentrations were higher in NTL of Peruvians. Importantly, metal concentrations were lower in HCC areas compared to NTL tissues in both cohorts, except for Cu for which mean concentration was higher in HCC (p < 0.05). Se concentration in HCC was associated with extended survival only in Peruvians. Our data, obtained in Peruvian and French HCC-NC cohorts, highlights similarity in the metallomic profile of HCC compared to NTL during the hepatic tumorigenesis in these specific groups of patients.

## Introduction

Hepatocellular carcinoma (HCC) is the most frequent primary liver cancer, the sixth most common cancer, and the third cause of death by cancer worldwide^1^. Many pathophysiological factors are potentially involved in the onset and progression of HCC, most of them being related to the chronic insult of the liver parenchyma. The development of HCC is commonly regarded as a sequential multistep pathogenic process initiated with inflammation-mediated liver tissue damages and hepatocyte necrosis that induce liver fibrogenesis towards cirrhosis, which in turn increases the risk for HCC^2^. Thus, a larger number of HCC cases reported in the literature are found in cirrhotic patients, whereas non-cirrhotic HCC (HCC-NC), is more rarely described.

We have previously described a peculiar clinical and molecular presentation of HCC-NC developed by patients from Peru^3^. These patients exhibited consistently remarkable clinical features: (a) 50% of them are relatively young with a median age below 40 including children, teenagers, and young adults; (b) the very large majority of the individuals presented with advanced-stage HCC and tumors larger than 10 cm in diameter; and (c) 90% of HCC occurred in non-cirrhotic liver^4^. This particular epidemiological context in which HCC-NC affects younger individuals was then corroborated to the whole region of South America^5^.

We further substantiated this peculiar presentation of HCC-NC at both molecular and histological levels. First, Peruvian HCC displayed a unique mutation spectrum, in which the major class of alterations was epitomized by genetic short indels^6^. Second, the integrative analysis revealed that Peruvian tumors correspond to a divergent molecular subtype of HCC with unique gene expression signature and global DNA hypermethylation pattern, albeit genome-wide hypomethylation was hitherto viewed as a hallmark of HCC^7^. Third, liver parenchyma exhibited a very low level of inflammatory response and an absence of fibrotic process, and that, despite a strong prevalence of underlying infection with hepatitis B virus^8,9^. However, we observed within the non-tumor liver (NTL) parenchyma the presence of foci of cellular alteration in which cells are smaller compared to regular hepatocytes and exhibit an altered nuclear-cytoplasmic ratio^8^. These foci of cellular alteration showed also some degree of congruence with the co-expression of glutamine synthetase. Altogether, these findings suggest that the clinical epidemiological situation encountered is due to some biological features intrinsic to the natural history of HCC in a fraction of the population in South America^10^. This observation prompted us to search for additional pathophysiological cofactors associated with HCC-NC that could enhance the risk of developing precociously HCC among Peruvian patients.

It has been reported that the toxic effects of metals and their role as a cofactor in the occurrence of HCC^11^. For example, increased hepatic iron stores have been associated with HCC-NC^12^. Oxidative stress and reactive oxygen species promoted by siderosis are strongly suggested to be instrumental in HCC. Furthermore, As has been classified as an enhancer of oxidative stress and a human carcinogen, notably for HCC^13^. However, other metals such as Mn, Se, and Zn are required for a normal activity of the antioxidant defense system in cells in a concentration-dependent manner^14^.

To evaluate relationships between the trace metal levels and the onset of early-age HCC-NC in Peru, we performed a comprehensive analysis of metal concentrations in both HCC and NTL tissues of Peruvian patients. In parallel, we contrasted these patients with another cohort of French individuals who developed HCC-NC in utterly different environmental, behavioral, and clinical contexts.

## Results

### Clinical and histological data

**Figure 1:**
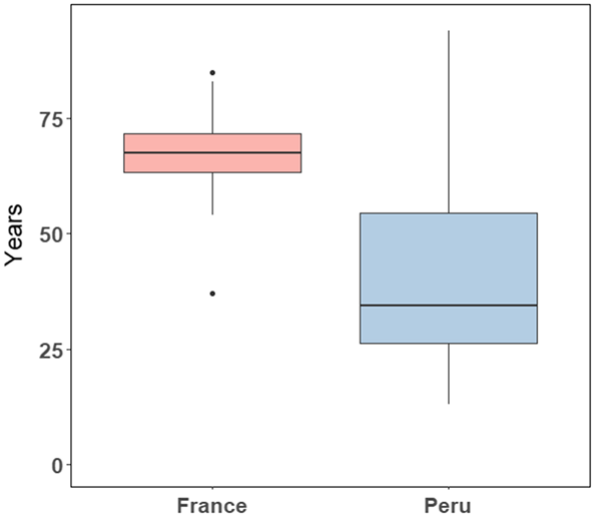
Age dispersion in both cohorts. Boxplot showing median, minimum, and maximum interquartile range (IQR) values for ages between Peruvian and French patients. Data were analyzed with Mann Whitney test (p < 0.001).

Table 1 summarizes the clinical and histopathological findings in both Peruvian and French cohorts. At the time of diagnosis, the mean age of the Peruvian patients was 40.6 ± 20.1 years old. A large component of young patients was found in the Peruvian cohort. On the other hand, the mean age for the French patients was 67.9 ± 9.3 years old (Fig. 1 and Supplementary Figure S1). The age difference at diagnosis was, thus, significantly different between both cohorts (*p* < 0.001).

**Table 1:** Main clinical features of patients included in the Peruvian and French cohorts.

Variable	French cohort N = 38	Peruvian cohort N = 38	*p*-value
**Age (years)**			< 0.001
Mean	67.5 (± 9.03)	40.6 (± 20.1)	
Range	[37 – 85]	[13 – 94]	
**Sex**			0.048
M	34	26	
F	4	12	
**Tumor size (cm)**			< 0.001
Mean	7.0 (± 5.09)	14.3 (± 5.1)	
Range	(1–20)	(4.50–27)	
**Hepatitis B virus**			< 0.001
Positive	2 (2.73%)	19 (26.02%)	
Negative	33 (45.20%)	19 (26.02%)	
**Fibrosis stage**			0.272
Absent	5	7	
Stage 1	9	12	
Stage 2	16	8	
Stage 3	8	11	
Stage 4	0	0	
**Histological grade**			0.016
Well differentiated	9	3	
Moderately differentiated	23	28	
Poorly differentiated	2	7	
Undifferentiated	4	0	
**Vascular invasion**			0.107
Yes	24	16	
No	14	22	

For numerical variables, mean values are presented with ± standard deviation (SD). For categorical variables, data are presented as number of cases. Levels of significance (*p* < 0.05) were calculated with Mann Whitney U-test.

The Peruvian cohort included 26 men (68.4%) and 12 women (31.6%), whereas the French cohort consisted of four women (10.5%) and 34 men (89.5%). The gender ratio was also significantly different between the Peruvian and French cohorts (*p* = 0.05). The presence of a positive hepatitis B serology (HBsAg +) in the Peruvian cohort was reported for 19 patients (50%), while in the French cohort only two patients (2.7%) were HBsAg + (*p* < 0.001) (Table 1). The mean size of tumors in Peruvian patients was twice that of the French (*p* < 0.001).

### Determination of hepatic metal concentrations

Metal concentrations in NTL for both cohorts are reported in Table 2 and supplementary data (Supplementary Figures S2 and S4 and Supplementary Table S1). Among the 12 metals quantified, concentrations of seven essentials (Co, Cu, Mn, Mo, Rb, Se, and Zn) were increased in the Peruvian cohort compared to the French one. Two out of three toxic metals concentrations (As, Cd) were also increased in Peruvians whereas Pb concentration was not different between cohorts. Arsenic was detected above the quantification limit only in some Peruvian NTL specimens, but not in any NTL of French patients. By contrast, Sn concentrations were higher in French NTL tissues compared to Peruvian ones. Relationships between metal concentrations in non-tumor tissue with clinical variables—gender, presence or absence of hepatitis B infection maker, presence or absence of hepatic fibrosis—were studied (Supplementary Tables S2 and S3). Regarding gender, in the French group, Co, Mn, Mo, Sn concentrations were higher in females (n = 4 only, vs 34 males), whereas no significant difference was between male and female in the Peruvian cohort. Peruvian patients exhibiting positive hepatitis B markers presented lower levels in Cd, Co, and Fe compared to negative ones. No significant difference was found in the French group, which comprise a very small number of positive patients (n = 2, only). Regarding fibrosis, compared to non-fibrotic patients, Mo concentration was significantly decreased in fibrotic patients of both cohorts. Mn concentration was lower only in fibrotic Peruvian patients whereas Co and Zn were lower in the French cohort only.

**Table 2:** Metal quantification in non-tumoral tissues from patients in both cohorts.

Metal (ug/gr)		Non-tumoral tissues		*p*-value
		French cohort N = 38	Peruvian cohort N = 38	
Arsenic (As)	Median IQR	0 0	0 0.29	< 0.01
Cadmium (Cd)	Median IQR	1.67 1.41	2.92 3.33	< 0.01
Cobalt (Co)	Median IQR	0.12 0.06	0.15 0.09	0.03
Copper (Cu)	Median IQR	15.60 9.82	25.20 12.60	< 0.01
Iron (Fe)	Median IQR	515.35 682.17	512.50 509.00	0.41
Manganese (Mn)	Median IQR	4.32 1.99	7.34 2.47	< 0.01
Molybdenum (Mo)	Median IQR	1.72 1.53	3.20 1.76	< 0.01
Lead (Pb)	Median IQR	0 0.15	0.09 0.16	0.20
Rubidium (Rb)	Median IQR	17.95 6.15	21.80 11.87	< 0.01
Selenium (Se)	Median IQR	1.61 0.69	1.94 0.83	0.01
Tin (Sn)	Median IQR	0.24 0.25	0.10 0.10	< 0.01
Zinc (Zn)	Median IQR	159.10 74.42	245.25 111.80	< 0.01

Data are presented as median and interquartile range (IQR). Levels of significance (*p* < 0.05) were calculated with Mann Whitney U-test.

Metals concentrations in HCC for both Peruvian and French cohorts are presented in Table 3, as well as Supplementary Figure S3 and Supplementary Table S4. As was found again in some HCC specimens from the Peruvian cohort; iron concentration values were higher in HCC from French patients in comparison to Peruvian patients (Supplementary Figures S2 and S5).

**Table 3:** Metal quantification in tumoral tissues between both cohorts.

Metal (ug/gr)		Tumoral tissues		*p*-value
		French cohort N = 38	Peruvian cohort N = 38	
Arsenic (As)	Median IQR	0 0	0 0.21	< 0.01
Cadmium (Cd)	Median IQR	0 0.43	0.26 0.55	0.13
Cobalt (Co)	Median IQR	0 0	0 0	0.32
Copper (Cu)	Median IQR	12.35 28.62	14 26.70	0.62
Iron (Fe)	Median IQR	216.95 318.32	116.00 131.25	< 0.01
Manganese (Mn)	Median IQR	2.01 3.03	1.18 2.45	0.11
Molybdenum (Mo)	Median IQR	0.80 0.99	0.49 0.59	0.07
Lead (Pb)	Median IQR	0 0.04	0 0.04	0.84
Rubidium (Rb)	Median IQR	15.75 7.62	15.50 9.62	0.77
Selenium (Se)	Median IQR	1.35 0.60	1.41 0.77	0.74
Tin (Sn)	Median IQR	0 0.10	0 0.03	0.29
Zinc (Zn)	Median IQR	91.15 38.42	83.40 31.72	0.39

Data are presented median and interquartile range (IQR). Levels of significance (*p* < 0.05) were calculated with Mann Whitney U-test.

**Figure 2:**
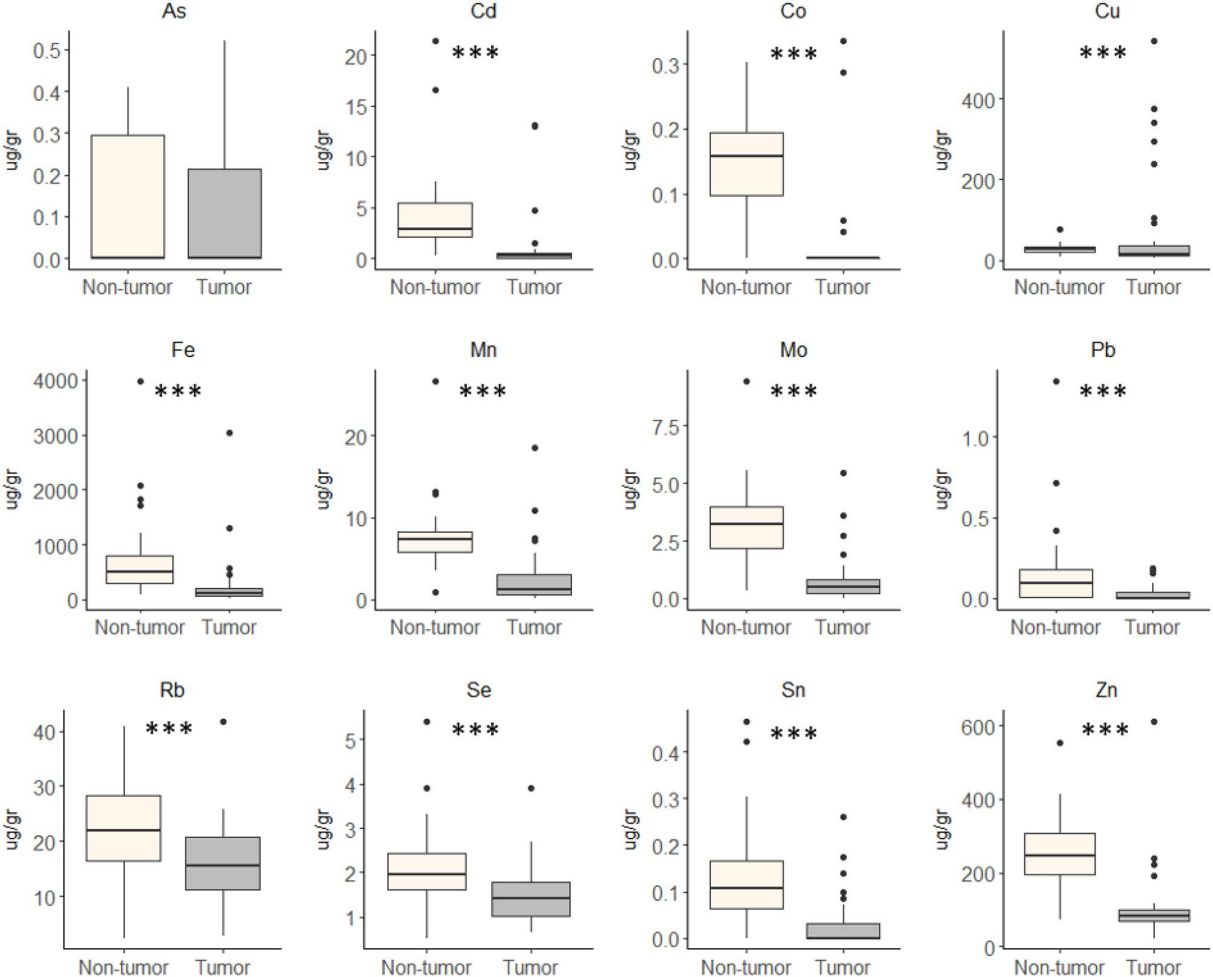
Comparison of metal concentration between tumoral and non-tumoral tissues in Peruvian cohort. Boxplot showing median, minimum, and maximum IQR values. Statistical test used was paired Mann–Whitney test.

**Figure 3:**
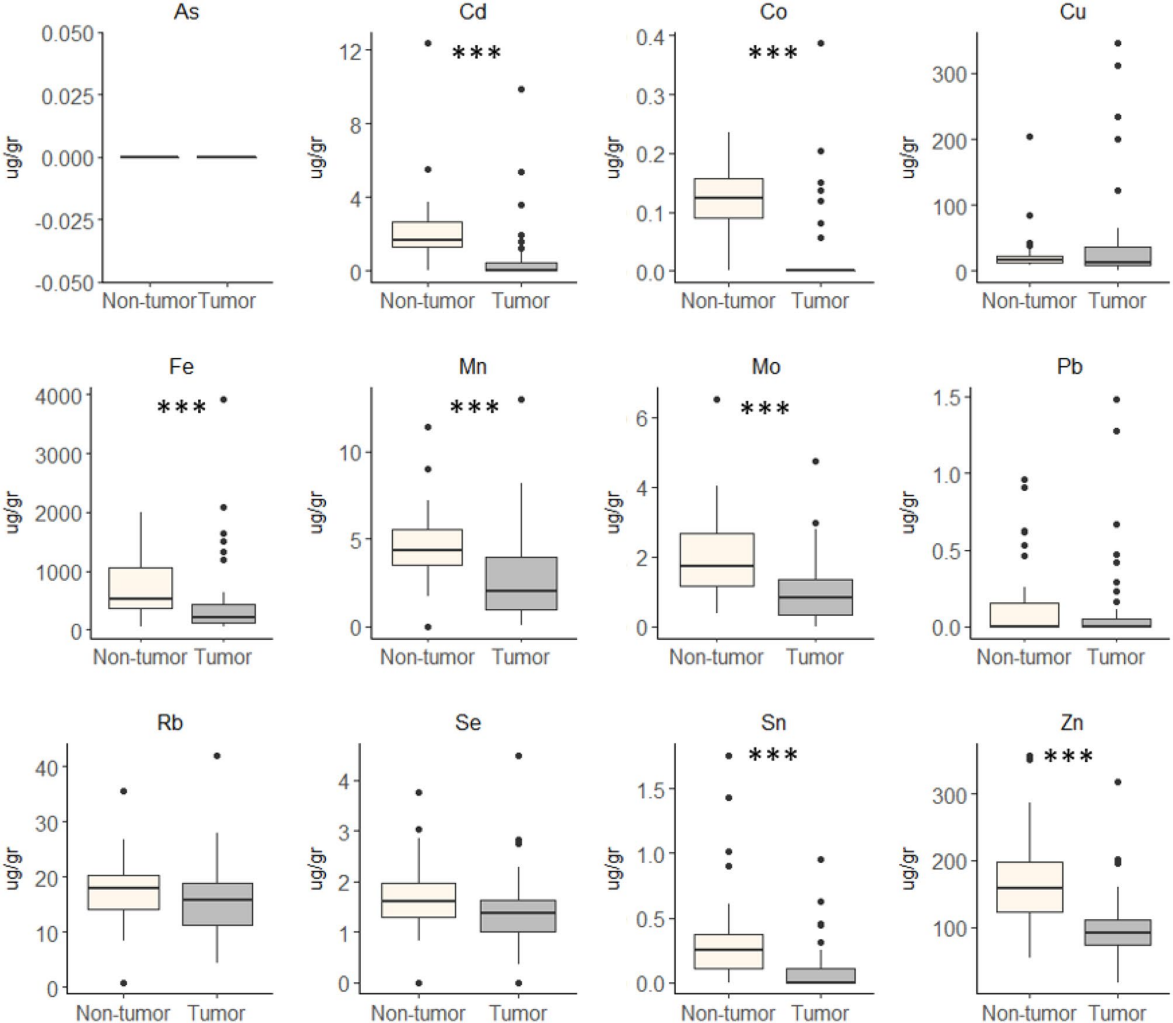
Comparison of metal concentration between tumoral and non-tumoral tissues in French cohort. Boxplot showing median, minimum, and maximum IQR values. Statistical test used was paired Mann–Whitney test.

In both cohorts, we found that metal concentrations were higher in NTL than in HCC (Figs. 2 and 3 and Supplementary Figure S3). The single exception was Cu that has higher concentrations in HCC than in NTL for the Peruvian cohort. In the French cohort, Cu concentrations were also slightly higher in HCC (49.4 ± 87.5 µg/gr) compared to NTL (25.3 ± 31.1 µg/gr), albeit without reaching the level of significance (*p* = 0.22).

### Survival analysis

The mean overall survival length in Peruvian patients was 65.7 weeks; whereas it was 148.4 weeks in French (*p* = 0.0071). A multivariate Cox proportional hazard ratio model was developed with all metal concentrations in both HCC and NTL. Our results showed that Se concentration in HCC samples had a protective effect (p < 0.04) (Supplementary Tables S5 and S6).

**Figure 4:**
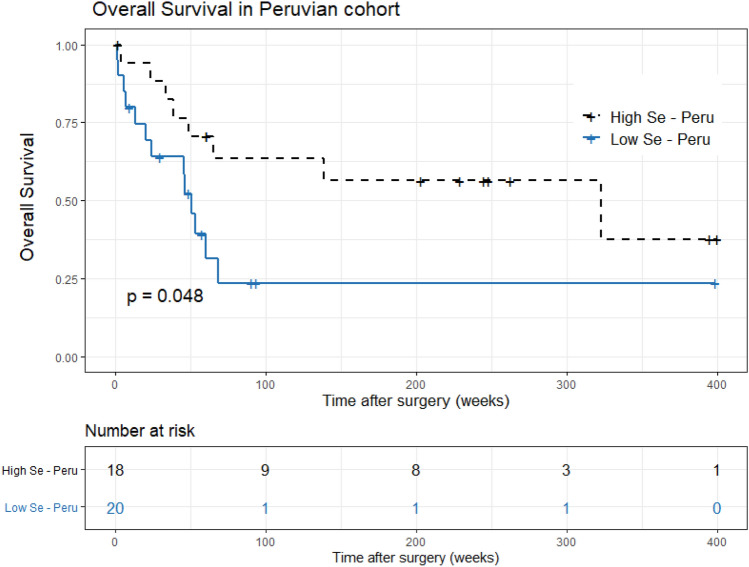
Survival plot in relation to selenium concentration in tumoral tissues in Peruvian cohort of patients. A significant decrease in survival duration is found for patients with low levels of Se in comparison to those with higher levels. Level significance (*p* < 0.05) was calculated using the Kaplan–Meier test.

Based on these results, we assessed the impact of Se on survival. To this aim, we stratified patients taking mean concentrations in HCC or NTL as cut-off values. Our data showed that Peruvian patients with tumor Se concentration above1.48 µg/g displayed a mean survival of 323 weeks versus 50 weeks for patients below this threshold (*p* < 0.048) (Fig. 4). We did not observe the corresponding partition in the French cohort (*p* = 0.31).

## Discussion

Our study presents, for the first time, a comparative analysis of liver metallomic profiles, in HCC and non-tumoral hepatic tissues, among patients from different geographical locations and genetic backgrounds, affected by HCC-NC. This disease diverges from the usual presentation of this tumor and represents at most 20% of HCC cases^15^. However, HCC-NC represents 90% of cases of liver cancer in the Peruvian context^3^. Considering the quantification of metals in tissue, it has been reported that cirrhosis may contribute to alter the metabolism of metals, such as iron, within non-tumor nodules and that it might exist some degree of heterogeneity between fibrotic and cellular areas in tissue. Then, a comparison between NC-HCC to HCC tissues may provide useful information to characterize cell metal content^16,17^.

The impact of metals on cell processes is a phenomenon not completely understood. As effectors of normal metabolism, essential metals are involved in many beneficial functions such as maintenance of pH, enzymatic cofactors, metabolic triggers, and reactive oxygen species formation^18–20^. However, when present in excess, metals may have harmful effects. The principal damaging impact of metals concerns the disruption of intracellular redox balance due to an increased reactive oxygen species production^21^.

Our data showed that concentrations of metals were higher in NTL of Peruvian patients compared to French individuals. Among those metals, some of them, such as As and Cd, are known to exert harmful effects on health and both are also considered carcinogenic to humans according to the WHO^22,23^. The mechanism by which As contributes to the process of carcinogenesis is DNA damage with chromosomal aberrations, deletion mutations, and aneuploidy^24,25^. A strong link between exposure to arsenic and the development of HCC has been demonstrated in animal models, which also evidenced an increase in lipid peroxidation levels, before the onset of the fibrosis process and subsequent development of HCC^24,26,27^. The mechanism of Cd related injury involves the interaction with thiol groups and also a possible inactivation of them, leading to functional alteration of the metalloenzymes of the superoxide dismutase family and subsequent depletion of antioxidant agents such as glutathione^28,29^.

It is important to mention that we cannot assess whether HCC-NC in Peruvian patients is directly caused by the presence of heavy metals^30^. Whether heavy metals play a role as enhancing factor in the carcinogenic process in association with hepatic carcinogenic agents, such as the hepatitis B virus infection in Peruvian patients or an excessive alcohol intake, could not be ascertained with sufficient confidence in both Peruvians and French cohorts^31^. To clarify this issue, studies should be conducted with a larger number of patients.

The most important highlight is the metallomic profile in both cohorts characterized by lower concentrations of metals in the tumor counterpart. Such finding suggests that whatever the etiological factors, the geographic origin, or any other parameters between cohorts, cancer cells develop similar adaptive processes regarding metal metabolisms. Whether the concentration decrease of most of these metals is related to a lower uptake, to an active elimination, and/or an increased turnover especially related to cell proliferation and metabolism remains unknown to date. However, the fact that metals are brought by alimentation, and conveyed by the portal vein to the non-tumor tissue while the essentially arterial vasculature of tumors is comparatively poorer in nutritional elements should also contribute to the observed differences.

The increase of Cu concentration, mostly in cancer tissues, was already reported in HCC^11^, and other cancers (breast, cervix, ovarian, and lung)^26,32,33^. A hypothesis aiming to explain the behavior of Cu in cancer has been proposed by Fisher and collaborators, who claimed that the increase of this metal is due to reduced catabolism of ceruloplasmin (Cp) in tumoral cells^34^. This phenomenon affecting the multicopper-carrying protein, might be due to its increased sialylation resulting from the presence of free sialic acid on the membranes of neoplastic cells. This hypothesis was later supported in an animal model by Bernacki et al.^35^. Another hypothesis concerns the role of copper as an angiogenic agent^36^. McAuslan et al*.* showed that copper acts as a promoter of endothelial cell migration^37^. Martin et al*.* consolidated both hypotheses, describing the link between Cu, ceruloplasmin, and HIF-1α^38^. The authors showed that Cu acts as a stabilizer of the HIF-1α, mediating inhibition of prolyl-4-hydroxylation. The HIF-1α is eventually responsible for regulating the transcription of many genes, including the Cp gene. Meanwhile, Himoto⁠ proposed a possible mechanism whereby Cu is required for binding HIF-1α to p300 and prevents the effect of Factor Inhibiting HIF-1 (FIH-1)^39^.

Another finding is the relationship between Se concentration in tumor tissues and survival in the Peruvian group. Se plays a major role in cell homeostasis, mainly through selenoproteins that are anti-inflammatory, chemo-preventive, and immune modulators^40^. This result is corroborated by a meta-analysis that confirmed the negative correlation between Se concentration and HCC development^41^. However, we did not reproduce this observation in the French cohort. This could be explained by the fact that, in Peruvian patients, a “natural evolution of the disease” is observed^4^, since they were treated only by surgery, whereas the French patients received additional local or systemic treatments that could modify HCC progression^42,43^. Therefore, the relationship between Se concentration in hepatic tumor and survival must be confirmed in larger cohorts integrating especially the type of adjuvant treatment to surgery.

Finally, we must highlight the possible role of the environment in the hepatocarcinogenic process due to the high mineral content of the subsoil and rivers. This statement was endorsed by several studies showing the presence of high heavy metal concentrations in the Andean regions of Peru^44–46^. In Egypt, Elwakil et al*.* have also described high concentrations of Cd, Pb, As, and Hg in blood samples from HCC patients who were exposed to the consumption of contaminated plants^47^. Therefore, we cannot rule out the relationship between the presence of metal in the environment and the natural history of HCC.

Altogether, our findings show that Peruvian and French cohorts of HCC patients have different metallomic profiles in NTLs, suggesting a putative impact of environmental and/or genetic factors. Whether these elements play a role in the very peculiar phenotype of HCC in Peru should be further explored. Moreover, the modulation of metal concentration profile in HCC, shared by the two cohorts, suggests a coordinated modulation of the metal metabolism in liver cancer cells during carcinogenesis. Importantly our data are obtained in a very specific group of patients that facilitate comparison between concentrations in tumoral and non-tumoral areas, cannot be directly extended to all HCC, especially those developed in a cirrhotic liver, that can also present inflammatory process. Additional studies will be required to improve our understanding of the relationship between metal metabolisms alterations and the hepatocarcinogenic process.

## Methods

### Ethical agreement

The present study investigated in strict accordance with the ethical principles contained in the Declaration of Helsinki. The Peruvian collection was approved by the Human Subjects Committee of the National Cancer Institute of Peru (INEN), Protocol Number INEN 10-05. Written informed consent was provided and signed by participants. When the patient was non-adult, a parent provided the informed consent on his/her behalf. For the French cohort, the biological material used as well as the clinical records data complied with the norms established by the European legislation (2001/20/EC) and the French Ethics Committee.

### Study design and patient selection

The present study was developed retrospectively in two cohorts, one Peruvian and one French. All patients included in the present study exhibited HCC-NC and were treated by surgical hepatic resection. For both cohorts, collected cryopreserved tissues and paraffin-embedded tissues of HCC and NTL were evaluated. Bioclinical parameters were collected from clinical records, unfortunately, alcohol intake was not extractable from medical records with certainty. Finally, follow-up data for survival analysis were obtained from the National Registry of Identification (RENIEC) in the case of the Peruvian cohort. For the French cohort, the information was obtained from the national obituary system.

Peruvian HCC-NC cohort consisted of 38 patients who were hospitalized at INEN between January 2010 and December 2016. None of these patients received preoperative treatment. The French cohort was initially made up of 45 patients hospitalized in the Rennes University hospital between January 2014 and March 2017. Patients who underwent preoperative treatment (n = 7) were then excluded from the French cohort and 38 patients were finally considered. Participants from both cohorts were selected on their histopathological report, considering the diagnosis of HCC-NC. For the Peruvian cohort, samples were obtained from the INEN Pathology Department and INEN Cancer Research Biobank. For the French cohort, samples were obtained from the Pathology department and Biological Resources Center in Rennes.

### Histological analysis

Formalin-fixed, paraffin-embedded tissues (FFPE) and hematoxylin–eosin (H&E) slides from tumoral and nontumoral areas were stained using the Masson’s Trichrome Stain Kit, Artisan™ (Dako), according to the manufacturer’s instructions. The stains were performed on the Histo-Pathology High Precision (H2P2) platform—ISO 9001 certified—UMS Biosit, within the University of Rennes 1, France.

Histopathological analysis of HCC comprises architecture and grading (G1-G4) according to the World Health Organization and the American Joint Committee on Cancer classifications, respectively ^48,49^. The liver fibrosis stage (0–4) was then scored following the scoring system for fibrosis and cirrhosis described by Scheuer and collaborators ^50^⁠. All histopathological parameters were independently evaluated by two pathologists (LC and BT). In case of divergence, a consensus was adopted.

### Trace elements quantification

All samples were treated to avoid environmental metal contamination. HCC and NTL samples were desiccated overnight at 120 °C. Then, dried tissues were weighed and mineralized by nitric acid solution in Teflon PFA-lined digestion vessels. Acid digestion was carried out at 180 °C using ultrapure concentrated HNO3 (69%) (Fisher Chemical Optima Grade) in a microwave oven device (Mars 6, CEM). The elements studied were either essential—manganese (^55^Mn), iron (^56^Fe), copper (^63^Cu), cobalt (^59^Co), zinc (^66^Zn), selenium (^78^Se), rubidium (^85^Rb), molybdenum (^96^Mo), or toxic -arsenic (^75^As), cadmium (^112^Cd), tin (^119^Sn), lead (^207^Pb). All these were measured by Inductively Coupled Plasma Mass Spectrometry (ICP-MS) in an X-Series II from Thermo Scientific equipped with collision cell technology (Platform AEM2, Biochemical Laboratory, Rennes 1 University—Rennes hospital). The source of plasma was argon (purity degree > 99.99%) ^51^.

The collision/reaction cell used was pressurized with a mixture of helium (93%) and hydrogen (7%); argon and hydrogen were provided by Messer. Ultra-pure water was provided from Millipore Direct-Q 3 water station. Nitric acid solution utilized at 69% (Fisher Chemical—Optima Grade). The rhodium was used like an internal standard (Fisher Scientific). Calibration ranges were realized using a multi-element solution (SCP Science Plasma Cal). The performance was calibrated using multi-element solutions, tune F, and tune A (Thermo). Certified reference material bovine liver ZC71001 was obtained from NCS Testing Technology (Beijing, China).

When a metal concentration was below the limit of quantification of the method, the value was arbitrary indicated as 0.

### Statistics

Data collected were inputted into Numbers software version 5.3 (Apple Corporation). All statistical analyses were performed in R version 3.5.1, "Feather Spray" (R Foundation). The comparative analyzes between both cohorts were performed using the Mann–Whitney test. The comparative analysis between tumor and non-tumor areas in each cohort was performed using paired Mann–Whitney test. For the survival analysis, the surgery date was considered as the starting parameter for the calculation of survival. Univariate Cox regression model was done with all variables ^52^. Later all variables with significative values were used in a multivariate Cox model, the resulting model was improved with backward and forward stepwise regression. Finally, the evaluation between all models was done with Akaike Information Criterion (AIC) index. The best model was selected from the lowest AIC value. Finally, for each metal, we used the concentration average to create groups, i.e. over and under the average, and to develop a comparative analysis of survival between these groups.

## Supplementary Information

Supplementary Information.

## Data Availability

Additional files (tables and graphs) are available as supplementary file. The datasets generated during and/or analyzed during the current study are available from the corresponding authors on reasonable request.
