# Supplementary material for: Metallomic profile in non-cirrhotic hepatocellular carcinoma supports a phenomenon of metal metabolism adaptation in tumor cells
Source: Sci Rep. 2021 Jul 9;11:14195. doi: 10.1038/s41598-021-93369-4 (PMC8271004; doi:10.1038/s41598-021-93369-4)
Supplement: Supplementary file 1 — Supplementary Information. [file 41598_2021_93369_MOESM1_ESM.docx]

**Supplementary data**


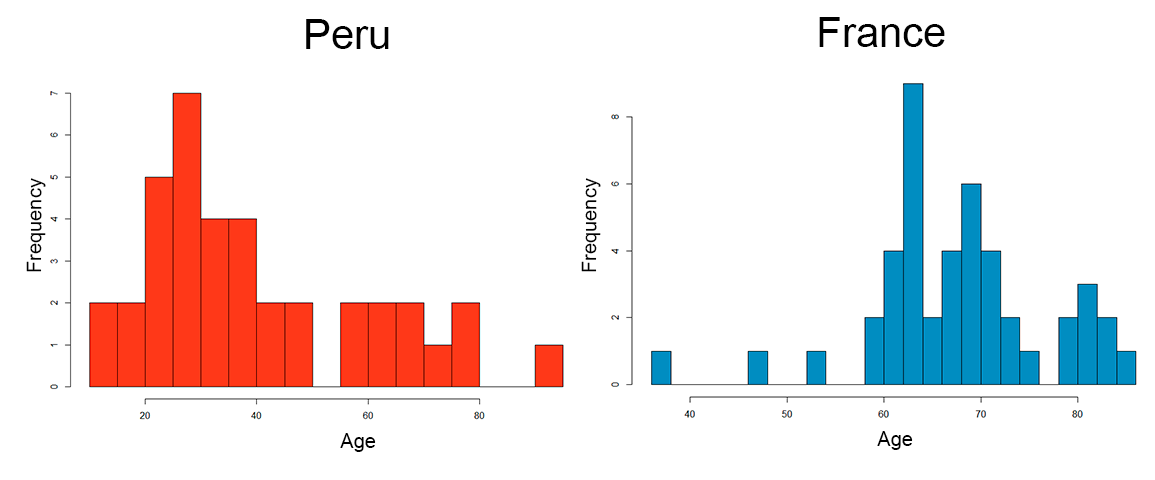


**Supplementary Figure S1: Histograms of age distribution in Peruvian and French cohorts.**


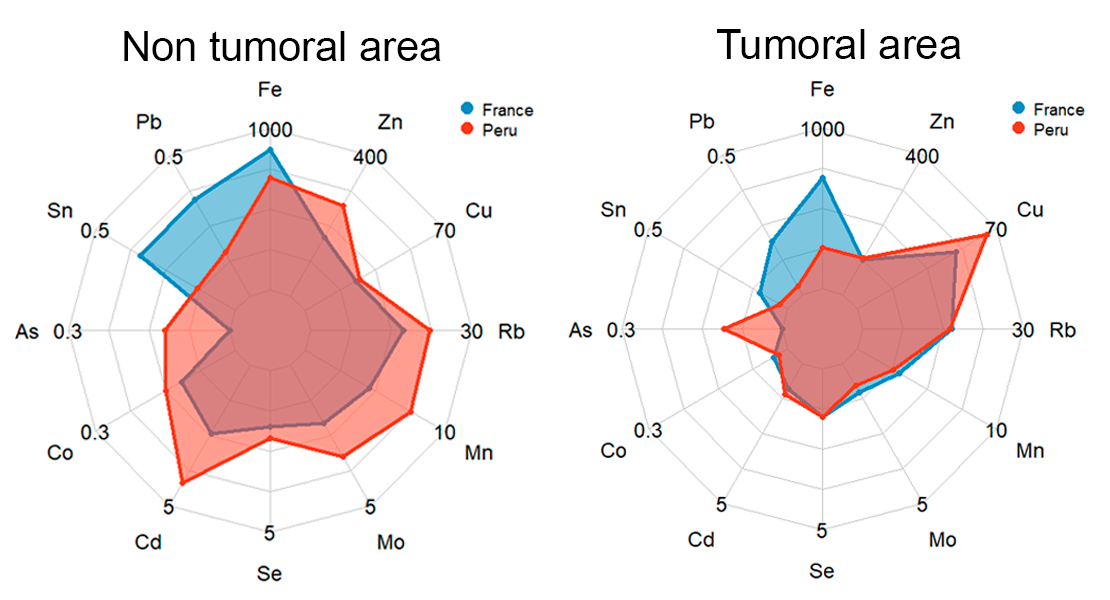


**Supplementary Figure S2: Radar charts of metallomic profile in tumoral and non-tumoral tissues.** Left panel: Metallomic profile for NTL. Toxic metals as As and Cd are higher in Peruvian cohort (red). However, Fe, Pb, and Sn are higher in French cohort (blue). Right panel: Metallomic profile for HCC. Fe, Pb, and Sn were higher in French cohort. As was exclusively quantifiable in Peruvian cohort.


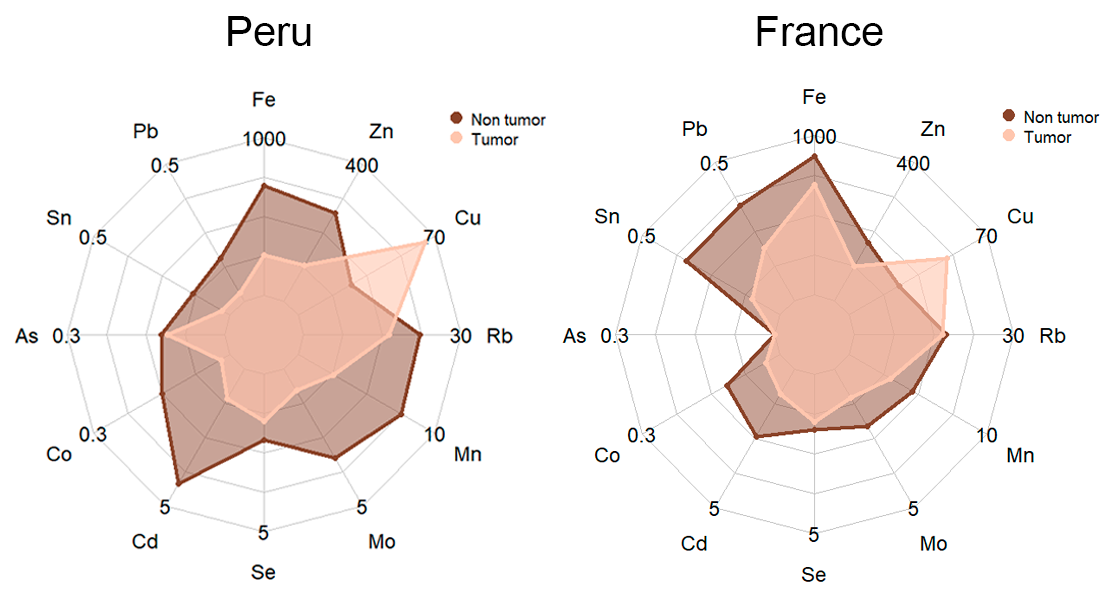


**Supplementary Figure S3: Comparative radar charts of metallomic profile in Peruvian and French cohorts.** Radar charts present in every corner a metal and its concentration (µg/g). In French (left panel) and Peruvians (right panel), the highest concentrations of metals were found in NTL (darker) except for Cu, which was the only one metal with higher concentrations in HCC (lighter) for both cohorts. As was found exclusively in Peruvian cohort.


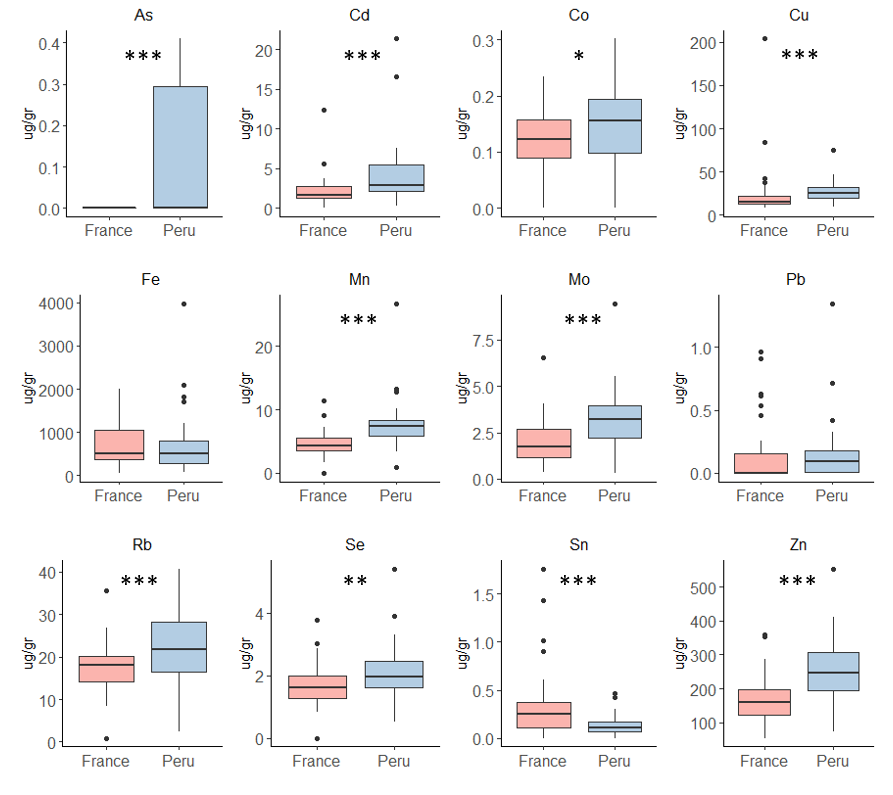


**Supplementary Figure S4: Metallomic profiles in non-tumoral liver tissues.** Boxplots showing metal concentrations in Peruvian and French cohorts. For As, when the values were under the limit of quantification for the method, they were arbitrary stated as 0. Values were normalized using percentile transformation for graphic purposes. Statistical test used was Man-Whitney test.


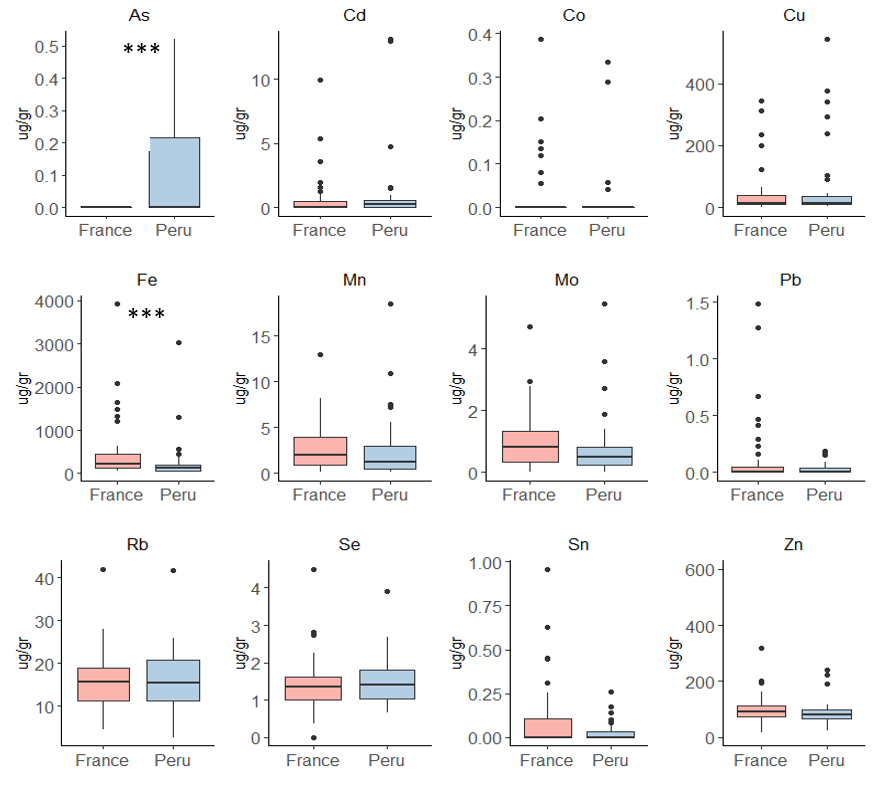


**Supplementary Figure S5: Metallomic profiles in tumoral liver tissues.** Boxplots showing differences in metal concentrations in Peruvian and French cohorts. For As, when the values were under the limit of quantification for the method, they were arbitrary stated as 0. Values were normalized using percentile transformation for graphical purposes. Statistical test used was Man-Whitney test.

.

| **Metal (µg/gr)** | **Non-tumoral tissues** | | ***p*-value** |
| --- | --- | --- | --- |
|  | **French cohort**  **Mean (±SD)**  **Range** | **Peruvian cohort**  **Mean (±SD)**  **Range** |  |
| **Arsenic (As)** | 0 | 0.12(±0.2)  [0.00 – 0.41 | **< 0.01** |
| **Cadmium (Cd)** | 2.4 (±2.7)  [0 – 13.3] | 4.20 (±4.1)  [0.2 – 21.3] | **<0.01** |
| **Cobalt (Co)** | 0.1 (±0.1)  [0 – 0.3] | 0.1 (±0.1)  [0 – 0.3] | **0.03** |
| **Copper (Cu)** | 25.3 (±31.1)  [6.9 – 204.1] | 27.3 (±12)  [9 – 75] | **0.01** |
| **Iron (Fe)** | 873.8 (±1024.9)  [56.2 – 6275.4] | 698.6 (±721.4)  [87 – 3973] | 0.41 |
| **Manganese (Mn)** | 4.6 (±2.4)  [0 – 12] | 7.54 (±4)  [0.9 – 26.7] | **< 0.01** |
| **Molybdenum (Mo)** | 2 (±1.3)  [0 – 6.5] | 3.3 (±1.5)  [0.3 – 9.4] | **< 0.01** |
| **Lead (Pb)** | 0.3 (±1.2)  [0 – 8] | 0.2 (±0.2)  [0 – 1.3] | 0.20 |
| **Rubidium (Rb)** | 17.2 (±6)  [0.9 – 35.6] | 22.15 (±8.5)  [2.4 – 40.7] | **<0.01** |
| **Selenium (Se)** | 1.7 (±0.7)  [0 – 4.1] | 2.1 (±0.8)  [0.5 – 5.4] | 0.01 |
| **Tin (Sn)** | 0.3 (±0.4)  [0 – 1.8] | 0.1 (±0.1)  [0 – 0.5] | **<0.01** |
| **Zinc (Zn)** | 167.6 (±68.9)  [46.2 – 385.5] | 258.1 (±103.3)  [71.9 – 553.7] | **< 0.01** |

**Supplementary Table S1: Metal concentration in non-tumoral tissues between Peruvian and French cohort.** Data are presented as mean ± SD and [range]. Levels of significance (*p* < 0.05) were calculated with Mann Whitney U-test.

| **Clinical parameters** | | **Measures of metal concentration in non-tumoral tissues in**  **Peruvian cohort** | | | | | | | | | | | |
| --- | --- | --- | --- | --- | --- | --- | --- | --- | --- | --- | --- | --- | --- |
|  |  | **As** | **Cd** | **Co** | **Cu** | **Fe** | **Mn** | **Mo** | **Pb** | **Rb** | **Se** | **Sn** | **Zn** |
|  |  | **Median of values (µg/gr)** | | | | | | | | | | | |
| **Gender** | **M (n = 26)**  **F (n = 12)** | 0.00  0.00 | 2.47  3.12 | 0.15  0.16 | 26.55  23.05 | 491.50  656.50 | 7.34  7.23 | 3.40  2.55 | 0.07  0.09 | 23.05  20.75 | 1.97  1.92 | 0.11  0.08 | 234.90  268.15 |
|  | ***p-value*** | *0.25* | *0.76* | *0.77* | *0.54* | *0.47* | *0.78* | *0.37* | *0.77* | *0.75* | *0.72* | *0.80* | *0.77* |
| **HBV Infection** | **Positive (n = 19)**  **Negative (n = 19)** |  | 2.17  4.28 | 0.12  0.18 | 24.00  26.00 | 319.00  733.00 | 7.34  7.44 | 2.82  3.74 | 0.06  0.12 | 21.10  25.60 | 1.71  2.01 | 0.09  0.11 | 205.70  272.80 |
|  | ***p-value*** | *0.94* | ***0.009*** | ***0.008*** | *0.71* | ***0.001*** | *0.60* | *0.20* | *0.16* | *0.16* | *0.35* | *0.55* | *0.29* |
| **Fibrosis** | **Present (n = 19)**  **Absent (n = 19)** | 0.00  0.00 | 2.31  3.89 | 0.13  0.18 | 23.70  27.20 | 569.00  487.00 | 6.33  7.68 | 2.35  3.57 | 0.07  0.10 | 21.80  21.20 | 2.09  1.89 | 0.10  0.11 | 229.20  273.40 |
|  | ***p-value*** | *0.59* | *0.51* | *0.09* | *0.53* | *0.75* | ***0.009*** | ***0.007*** | *0.17* | *0.78* | *0.60* | *0.95* | *0.29* |

**Supplementary Table S2: Relationships between metals and clinical parameters in non-tumoral tissues in the Peruvian cohort.**

| **Clinical parameters** | | **Measures of metal concentration in non-tumoral tissues in**  **French cohort** | | | | | | | | | | | |
| --- | --- | --- | --- | --- | --- | --- | --- | --- | --- | --- | --- | --- | --- |
|  |  | **As** | **Cd** | **Co** | **Cu** | **Fe** | **Mn** | **Mo** | **Pb** | **Rb** | **Se** | **Sn** | **Zn** |
|  |  | **Median of values (µg/gr)** | | | | | | | | | | | |
| **Gender** | **M (n = 34)**  **F (n = 4)** | NA  NA | 1.57  4.12 | 0.11  0.17 | 15.10  18.45 | 478.25  637.30 | 4.05  5.55 | 1.53  2.93 | 0.00  0.13 | 18.00  15.10 | 1.54  1.70 | 0.21  0.67 | 157.70  190.40 |
|  | ***p-value*** | *NA* | *0.08* | ***0.01*** | *0.44* | *0.70* | ***0.05*** | ***0.02*** | *0.34* | *0.61* | *0.41* | ***0.008*** | *0.16* |
| **HBV Infection** | **Positive (n = 2)**  **Negative (n = 33)** | 0.00  0.00 | 2.16  1.67 | 0.18  0.12 | 19.15  15.50 | 991.35  551.30 | 5.29  4.29 | 2.98  1.56 | 0.02  0.08 | 15.50  18.00 | 2.24  1.62 | 0.88  0.24 | 227.40  158.10 |
|  | ***p-value*** | *NA* | *0.83* | *0.08* | *0.52* | *0.52* | *0.43* | *0.11* | *0.49* | *0.39* | *0.39* | *0.83* | *0.15* |
| **Fibrosis** | **Present (n = 24)**  **Absent (n = 14)** | 0.00  0.00 | 1.65  2.50 | 0.11  0.14 | 15.10  16.85 | 458.75  672.10 | 4.01  4.55 | 1.47  2.52 | 0.00  0.02 | 18.05  17.90 | 1.54  1.64 | 0.23  0.29 | 134.85  204.85 |
|  | ***p-value*** | *NA* | *0.18* | ***0.03*** | *0.58* | *0.14* | *0.15* | ***0.04*** | *0.84* | *0.93* | *0.34* | *0.28* | ***0.005*** |

**Supplementary Table S3: Relationships between metals and clinical parameters in non-tumoral tissues in the French cohort.**

| **Metal (µg/gr)** | **Tumoral tissues** | | ***p*-value** |
| --- | --- | --- | --- |
|  | **French cohort**  **Mean (±SD)**  **Range** | **Peruvian cohort**  **Mean (±SD)**  **Range** |  |
| **Arsenic (As)** | 0 | 0.1 (±0.1)  [0 – 0.5] | **< 0.001** |
| **Cadmium (Cd)** | 0.9 (±2)  [0 – 9.9] | 1.1 (±3)  [0 – 13.1] | 0.13 |
| **Cobalt (Co)** | 0.03 (±0.1)  [0 – 0.4] | 0.02 (±0.1)  [0 – 0.3] | 0.32 |
| **Copper (Cu)** | 49.4 (±87.5)  [0 – 345.2] | 65 (±123.7)  [4 – 543.2] | 0.62 |
| **Iron (Fe)** | 689.6 (±1538)  [55.4 – 9941] | 255.9 (±511.7)  [24 – 3026] | **< 0.01** |
| **Manganese (Mn)** | 3 (±2.9)  [0.1 – 13] | 2.6 (±3.6)  [0.1 – 18.5] | 0.11 |
| **Molybdenum (Mo)** | 1 (±1)  [0 – 4.7] | 0.8 (±1)  [0 – 5.4] | 0.07 |
| **Lead (Pb)** | 0.2 (±0.5)  [0 – 2.7] | 0.03 (±0.05)  [0 – 0.2] | 0.84 |
| **Rubidium (Rb)** | 16.6 (±7)  [4.4 – 42] | 16.2 (±8.8)  [2.7 – 41.8] | 0.77 |
| **Selenium (Se)** | [1.5 (±0.8)  [0 – 4.5] | 1.5 (±0.7)  [0.7 – 3.9] | 0.74 |
| **Tin (Sn)** | 0.1 (±0.2)  [0 – 1] | 0.03 (±0.1)  [0 – 0.3] | 0.29 |
| **Zinc (Zn)** | 99.4 (±53.9)  [17.4 – 318.9] | 104.6 (±96.3)  [23.4 – 612.1] | 0.39 |

**Supplementary Table S4: Metal concentration in tumoral tissues between Peruvian and French cohort.** Data are presented as mean ± SD and [range]. Levels of significance (*p* < 0.05) were calculated with Mann Whitney U-test.

| **Metal (µg/gr)** | **Cox regression in tumor tissues for Peruvian cohort** | | ***p*-value** |
| --- | --- | --- | --- |
|  | **HR** | **CI** |  |
| **Arsenic (As)** | 1.01 | [1.01–0.06] | 0.99 |
| **Cadmium (Cd)** | 0.93 | [0.73–1.19] | 0.57 |
| **Cobalt (Co)** | 0.02 | [0.00–3847.26] | 0.53 |
| **Copper (Cu)** | 1.00 | [0.99–1.00] | 0.47 |
| **Iron (Fe)** | 1.00 | [1.00–1.00] | 0.56 |
| **Manganese (Mn)** | 0.89 | [0.74–1.08] | 0.24 |
| **Molybdenum (Mo)** | 0.39 | [0.14–1.11] | 0.08 |
| **Lead (Pb)** | 202.43 | [0.1–6489.46] | 0.32 |
| **Rubidium (Rb)** | 0.97 | [0.92–1.03] | 0.32 |
| **Selenium (Se)** | 0.39 | [0.16–0.95] | **0.04** |
| **Tin (Sn)** | 1.77 | [0.00–4592.64] | 0.89 |
| **Zinc (Zn)** | 0.99 | [0.98–1.00] | 0.13 |

**Supplementary Table S5: Cox regression coefficients values for metals in HCC tissues of Peruvians patientss.**

| **Metal (µg/gr)** | **Cox regression in tumor tissues for French cohort** | | ***p*-value** |
| --- | --- | --- | --- |
|  | **HR** | **CI** |  |
| **Arsenic (As)** | NA | NA | NA |
| **Cadmium (Cd)** | 0.81 | [0.41–1.60] | 0.55 |
| **Cobalt (Co)** | 0.02 | [0.00–21808.45] | 0.60 |
| **Copper (Cu)** | 1.00 | [0.99–1.00] | 0.61 |
| **Iron (Fe)** | 1.00 | [1.00–1.00] | 0.26 |
| **Manganese (Mn)** | 0.59 | [0.36–0.99] | **0.05** |
| **Molybdenum (Mo)** | 0.33 | [0.09–1.18] | 0.09 |
| **Lead (Pb)** | 0.00 | ]0.00] | 0.99 |
| **Rubidium (Rb)** | 0.91 | [0.81–1.03 | 0.13 |
| **Selenium (Se)** | 0.35 | [0.11–1.06] | 0.06 |
| **Tin (Sn)** | 2.51 | [0.12–52.12] | 0.55 |
| **Zinc (Zn)** | 0.99 | [0.97–1.01] | 0.22 |

**Supplementary Table S6: Cox regression coefficients values for metals in HCC tissues of French patients.**
